# Enhancing Neuroblastoma Immunotherapies by Engaging iNKT and NK Cells

**DOI:** 10.3389/fimmu.2020.00873

**Published:** 2020-05-08

**Authors:** Kevin O. McNerney, Spyridon A. Karageorgos, Michael D. Hogarty, Hamid Bassiri

**Affiliations:** ^1^Division of Oncology, Children's Hospital of Philadelphia, Philadelphia, PA, United States; ^2^School of Medicine, European University Cyprus, Nicosia, Cyprus; ^3^Division of Infectious Diseases, Children's Hospital of Philadelphia, Philadelphia, PA, United States

**Keywords:** invariant natural killer T cells, natural killer cells, cancer immunotherapy, immunotherapy, neuroblastoma, tumor microenvironment

## Abstract

Neuroblastoma (NB) is the most common extracranial solid tumor in children and, in the high-risk group, has a 5-year mortality rate of ~50%. The high mortality rate and significant treatment-related morbidities associated with current standard of care therapies belie the critical need for more tolerable and effective treatments for this disease. While the monoclonal antibody dinutuximab has demonstrated the potential for immunotherapy to improve overall NB outcomes, the 5-year overall survival of high-risk patients has not yet substantially changed. The frequency and type of invariant natural killer T cells (iNKTs) and natural killer cells (NKs) has been associated with improved outcomes in several solid and liquid malignancies, including NB. Indeed, iNKTs and NKs inhibit tumor associated macrophages (TAMs) and myeloid derived suppressor cells (MDSCs), kill cancer stem cells (CSCs) and neuroblasts, and robustly secrete cytokines to recruit additional immune effectors. These capabilities, and promising pre-clinical and early clinical data suggest that iNKT- and NK-based therapies may hold promise as both stand-alone and combination treatments for NB. In this review we will summarize the biologic features of iNKTs and NKs that confer advantages for NB immunotherapy, discuss the barriers imposed by the NB tumor microenvironment, and examine the current state of such therapies in pre-clinical models and clinical trials.

## Introduction

Neuroblastoma (NB) is the most common extracranial solid tumor in children and accounts for ~15% of childhood cancer deaths (1). NB is stratified into risk groups on the basis of clinical and molecular features using the International Neuroblastoma Risk Group (INRG) classification with the high-risk group being the most prevalent (2). High risk neuroblastoma (HRNB) is treated with a combination of conventional chemotherapy, surgical resection, autologous stem-cell transplant, radiation, and immunotherapy. Despite the extensive treatment regimen, HRNB still carries a 5-year overall survival (OS) of ~50%, and these treatments have significant late adverse effects including hearing loss, cognitive deficits, endocrinopathies, and ovarian failure (1, 3–5). As such, there is a critical need for more tolerable and effective treatments in this group.

Immunotherapy with dinutuximab, a monoclonal antibody against GD2 (a disialoganglioside that is highly expressed on neuroblasts), has been incorporated into the recommended treatment regimen for HRNB after FDA-approval in 2015, and significantly improved 2-year event-free-survival (EFS) (6). Additionally, the addition of dinutuximab to irinotecan and temozolomide in the relapsed or refractory NB setting demonstrated dramatic clinical activity. In those treated with the dinutuximab-containing regimen, 9 out of 17 patients (53%) had a disease response vs. 1 out of 18 patients (6%) in a comparator arm that included the same chemotherapy but without dinutuximab (7). These studies demonstrate that dinutuximab immunotherapy has significant clinical utility in both minimal and high disease burden contexts. However, despite the improved 2-year event-free-survival (EFS) in the minimal residual disease setting and substantial response in the bulk disease setting, 5-year overall survival (OS) in HRNB remains ~50%. Improving the long-term efficacy of these promising immunotherapies in HRNB therefore remains an important unsolved challenge.

While the use of chimeric antigen receptor (CAR) T cells and checkpoint inhibitors have shown success in the treatment of other malignancies, these immunotherapies have not yet demonstrated similar efficacy in HRNB or other pediatric solid tumors (8–10). The lack of response in HRNB is thought to be due to multiple factors including an immunosuppressive tumor microenvironment (TME), low expression of MHC-I, and MHC-II antigens on neuroblasts leading to immune evasion, low mutational burden in NB with a paucity of neoantigens, and diminished T cell persistence (for CAR T cell therapies) (11, 12). Alternative immunotherapies that can overcome these barriers are therefore being sought.

Interestingly, the presence of invariant natural killer T cells (iNKTs), and natural killer cells (NKs) is associated with improved prognosis of patients with NB as well as other malignancies (12–15). iNKTs and NKs inhibit tumor-associated macrophages (TAMs) and myeloid derived suppressor cells (MDSCs), kill cancer stem cells (CSCs), and tumor cells that have downregulated their MHC antigens, and robustly secrete cytokines to recruit additional immune effectors. These features allow NKs and iNKTs to decrease tumor immunosuppression and overcome immune evasion in the HRNB TME (16). Thus, NK and iNKT therapies may prove useful in both stand-alone and combination treatments for NB and other pediatric solid tumors.

## Biology of INKT Cells

### Overview and Relevance in Human Cancer

Natural Killer T cells (NKTs) are innate-like lymphocytes that make up about 1% of total lymphocytes in the human liver, and also have residence in the spleen and bone marrow. They bridge the innate and adaptive immune systems, helping to coordinate robust responses to malignant or infected cells, and have demonstrated importance in tumor immunosurveillance (17–19). NKTs share features with both NKs and T cells, and are divided into type I, or invariant NKTs (iNKTs), that express a conserved T cell receptor (TCR) made up of an α chain composed of Vα24 and Jα18 segments paired with a β chain composed of the Vβ11 segment. Conversely, type II NKTs express polyclonal TCRs, similar to conventional CD4+ and CD8+ T cells. These invariant TCRs allow for the recognition of glycolipid antigens (GAgs) presented by a non-polymorphic and conserved MHC class 1-like protein called CD1d. Due to the availability of CD1d tetramers specific for the invariant TCR, the majority of the literature on NKTs has focused on the role of iNKTs in antitumor responses; we will similarly do so in this review.

The frequency of iNKTs within a tumor, or in circulation, has been associated with improved survival and reduced progression in various malignancies including prostate cancer, medulloblastoma, melanoma, multiple myeloma, colon cancer, lung cancer, breast cancer, head and neck squamous cell carcinomas (HNSCC), and NB (12, 20, 21). Additionally, lack of function in iNKTs is associated with advanced cancers and worse prognosis in patients with multiple myeloma, myelodysplastic syndrome, and prostate cancer (22–25). In xenograft models for NB, mice lacking iNKTs had more metastases and shortened survival in comparison to their iNKT-replete counterparts (26). Additionally, in mice lacking one allele of the tumor suppressor p53, absence of iNKTs predisposed to earlier development of a variety of cancers and decreased survival (27). Finally, for NB patients at the time of diagnosis, a high frequency of iNKTs in NB tumors was found to be associated with improved survival and lower stage NB (28). Given the apparent importance of iNKTs in tumor immunology, elucidating the various modes of iNKT activation, and activity is of great interest.

### iNKT Activation and Activity

**Figure 1:**
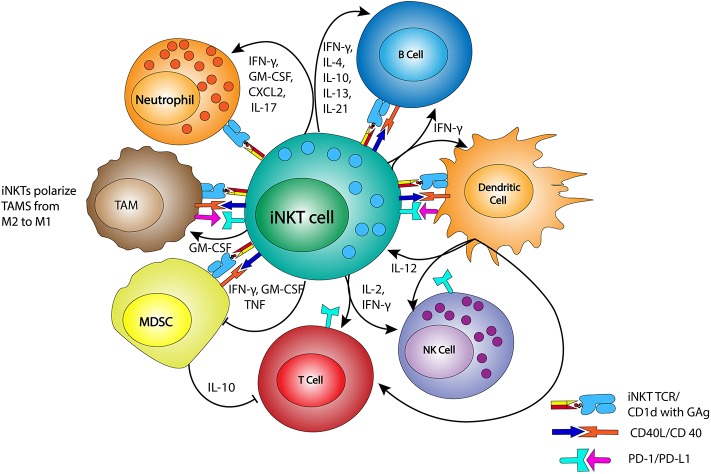
iNKTs and interactions with immune effectors. iNKTs interact with members of the immune system, including tumor associated macrophage (TAM), myeloid derived suppressor cells (MDSC), neutrophils, NKs, T cells, and B cells. These interactions occur via their T cell receptor (TCR) binding to CD1d complexes bearing glycolipid antigens (GAg), through CD40/CD40L binding, and/or through cytokine release. Dendritic cells are matured by iNKTs via CD40L/CD40 binding and the release of IFN-γ, which then stimulates IL-12 release and the reciprocal stimulation of iNKTs. IFN-γ and IL-12 also stimulate nearby NKs, T cells, and B cells, helping to generate a pro-inflammatory immune environment. TAMs are reprogrammed from M2 macrophage to more pro-inflammatory M1 macrophage by GM-CSF release. MDSC modulation by iNKTs reduces the inhibition of T cell function.

iNKTs can be activated in CD1d-dependent and -independent manners (Figure 1). As mentioned above, the iNKT TCR binds to GAg presented by CD1d proteins, which are expressed by most cells of hematopoietic origin including TAMs and MDSCs. Cells that are transformed or infected present immunogenic GAgs, or have changes in their actin cytoskeleton that create CD1d nanoclusters of high avidity, leading to iNKT activation (29–31). Independent of their CD1d-driven activation, iNKTs can also be activated through exposure to the cytokines IL-12 and IL-18 (32–34). Additionally, co-stimulatory signals from CD28, 4-1BB (CD137), NKG2D, CD40L, and ICOS (CD278) mediate robust iNKT activation (35–39). Dendritic cells (DCs) use cytokine release (IL-12) and cell-cell signaling with iNKTs through GAg-CD1d/TCR and CD40L/CD40 binding, and are of particular importance for iNKT activation. Notably, the production of cytokines IFN-γ and IL-12 by the activated iNKTs and DCs results in enhanced NK- and T cell mediated antitumor responses (12, 40–42).

When iNKTs are activated, they release large amounts of cytokines that mature, recruit, and activate other immune effector cells. Similar to conventional CD4+ T cells, iNKTs produce Th1-, Th2-, and Th17-like cytokines. Th-1 cytokines are associated with pro-inflammatory responses and enhanced antitumor cytotoxicity, whereas Th-2 cytokines promote immune tolerance and are useful in limiting autoimmunity (43). The avidity and stability of the CD1d-GAg complex influence the ratio of Th1:Th2 cytokines released (43–45). Furthermore, CD1d-GAg complexes with high affinity for the iNKT TCR have been shown to limit accumulation of immunosuppressive MDSCs in tumors when compared with lower affinity CD1d-GAg complexes. Higher affinity CD1d-GAg complexes also induce less iNKT anergy, which is defined by a lack of activation on repeated stimulation (45, 46). These observations illustrate the importance of the GAg in mediating iNKT stimulation+ (45, 46).

In addition to iNKT cytokine production, iNKTs can also recognize, kill, and reprogram CD1d+ cells including immunosuppressive TAMs and MDSCs. They mediate cytotoxic responses through release of perforin/granzyme B, or through upregulation of Fas ligand (FasL; CD178) and TNF-related apoptosis inducing ligand (TRAIL; CD253) (17, 42). iNKTs also produce granulocyte-macrophage colony stimulating factor (GM-CSF), which reprograms TAMs to display a pro-inflammatory (M1) phenotype (26, 47, 48). The ability to kill or reprogram TAMs is crucial, as expression of TAM-specific genes in the NB TME is associated with poor 5-year EFS (48). TAMs are not only immunosuppressive, but also promote neuroblast growth and increased osteoclastic activity associated with bone metastases through release of IL-6 (48, 49). Notably, iNKTs may be the only known effector cells that recognize and negatively regulate TAMs (50). iNKTs are also capable of culling IL-10 expression from immunosuppressive MDSCs, which can limit suppression of cytotoxic T cells, resulting in improved tumor control (51).

### Anergy and Tumor Microenvironment Immunosuppression of iNKT Cells

Despite their many favorable features, certain barriers face the use of iNKTs as cancer immunotherapeutics. One such limitation is that of anergy, a state in which iNKTs fail to produce cytokines, or proliferate after stimulation. Indeed, murine iNKTs have been shown to become anergic for periods of up to 6 months after a single exposure to α-GalactosylCeramide (α-GalCer), the highly potent canonical GAg used to stimulate iNKTs in numerous studies (46). iNKT anergy not only limited anti-tumor activity, but further stimulation of anergic iNKTs actually worsened tumor metastasis in a mouse model of melanoma (46).

Similar to conventional T cells, an additional barrier can be imposed by proteins expressed on neuroblasts, TAMs, MDSCs, and regulatory T cells (T_REG_). Proteins such as programmed cell death ligand-1 (PD-L1) binds to PD1 on iNKTs and other immune effectors to inhibit their cytotoxic function (52–57). Aside from expression of checkpoint ligands such as PD-L1, neuroblasts, and co-opted immune cells also release TGF-β1, IL-4, IL-6, IL-10, IL-13, adenosine, and prostaglandin E-2 to suppress infiltrating immune cells (48, 49). This immunosuppressive milieu can bias iNKTs toward Th2 cytokine release, thereby skewing the TME in an immunosuppressive direction.

These barriers have been targeted using strategies to reduce anergy and block checkpoint pathway signaling. Anergy has been shown to be inhibited by pulsing DCs with GAg, or loading soluble CD1d with synthetic GAgs modified for greater CD1d/TCR affinity. Indeed, the C-glycoside analog of α-GalCer and phenylated GAgs activate and skew iNKTs toward Th1-cytokine secretion, thereby counteracting the immunosuppressive cytokines of the TME (45). Phenylated GAgs also limit the anergy experienced by iNKTs on repeated stimulation and result in less accumulation of MDSCs in the TME than α-GalCer, suggesting the advantage of their use for iNKT stimulation (45). Finally, the immunosuppressive effects of checkpoint receptor expression have been targeted by antibody-mediated blockade of PD-1/PD-L1 interactions; this blockade restored IFN-γ release and augmented anti-tumor activity of iNKTs (37, 54, 56–58).

In summary, iNKTs are associated with favorable prognosis in various human malignancies, likely due to their ability to secrete pro-inflammatory cytokines and culling and/or reprogramming of immunosuppressive and tumor-growth promoting cells in the TME. As such, therapies that minimize iNKT anergy and promote the release of Th1 over Th2 cytokines are likely to have greater antitumor efficacy. We will now discuss iNKT-based treatments for NB that have been studied to date.

## INKT Cell-Based Treatments of Neuroblastoma

**Figure 2:**
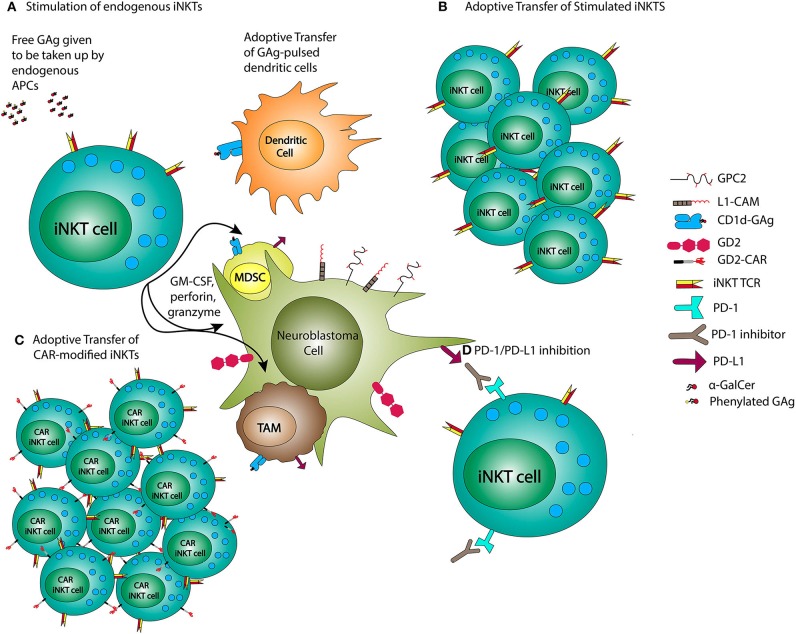
iNKT-based NB treatments. NB therapies using iNKTs may employ administration of glycolipid antigens (GAg) or GAg-pulsed dendritic cells **(A)**, adoptive transfer of previously stimulated iNKTs **(B)**, or CAR-modified iNKTs **(C)**, and combined with PD-1/PD-L1 inhibition **(D)**.

There has been an accumulation of pre-clinical and early phase clinical data indicating that iNKT-based immunotherapies have promise in the treatment of NB. The approaches employing iNKTs for immunotherapy of NB include GAg stimulation of iNKTs, adoptive transfer of iNKTs, and CAR-iNKTs (Figure 2).

### GAg Stimulation of iNKT Cells

GAg stimulation of iNKTs for antitumor therapy involves providing GAg alone or in the context of antigen-presenting cells (APCs) for promotion of antitumor immunity by endogenous iNKTs. This strategy has been used in patients with various solid tumors with modest, but encouraging results. For example, in a Phase I trial evaluating the effects of iNKT stimulation with α-GalCer in patients with advanced solid tumors, serum GM-CSF, and TNF-α were increased in 5 of 24 patients treated, albeit without disease response. Significantly, the patients that had a cytokine bump from the α-GalCer had higher levels of pre-treatment iNKTs, and there was no dose-limiting toxicity (59). Another strategy for GAg-stimulation of iNKTs involves pulsing DCs with α-GalCer, and administering these pulsed DCs intravenously. In various trials of patients with non-NB solid tumors, this strategy led to decreases in tumor markers, iNKT infiltration into tumors, and in some patients, tumor necrosis (60–62). In NB, iNKTs stimulated with CD1d–loaded with α-GalCer were shown to enhance NK-mediated killing of NB cell lines, but could not directly induce such killing when cell lines were CD1d–, as is characteristic of NB (63). However, in elegant co-culture studies, α-GalCer-activated iNKTs have been shown to enhance the NK antibody-dependent cellular cytotoxicity (ADCC) of NB cell lines when anti-GD2 antibodies were provided (64). In addition to enhancing NK-cell killing, iNKTs can reinvigorate exhausted CD8+ T cells. For instance, in melanoma, α-GalCer-pulsed APCs were administered to humanized mice xenografted with PD-1 inhibitor resistant melanoma. This restored cytotoxic activity of exhausted CD8+ T lymphocytes via IL-2 and IL-12 production, and resulted in reduced tumor progression and improved survival (65). iNKTs are therefore shown to be capable of enhancing an immune response to CD1d– tumors, such as NB, when activated with GAg or GAg-loaded APCs. However, given that many metastatic solid tumors including NB are associated with lower levels of iNKTs (limiting the potential benefits of mere iNKT activation for these patients), options for iNKT therapies that boost iNKT numbers *and* activation have been sought.

### Adoptive Transfer of iNKT Cells

Adoptive transfer of iNKTs has been attempted in numerous pre-clinical and clinical studies in NB and other solid tumors. The importance of iNKTs in tumor immunity in NB was demonstrated in iNKT-deficient and iNKT-replete mice xenografted with NB, with the iNKT-replete mice developing significantly fewer metastases and having longer survival than iNKT-deficient mice (26). When iNKTs were adoptively transferred to humanized NSG mice with NB xenografts, TAMs were reprogrammed from M2 to the M1 phenotype. Despite this reprogramming, NB tumors progressed, and adoptive transfer of iNKTS resulted in increased PD-L1 expression on M1 and M2 TAMs (66). Given that iNKTs increase their PD1 expression on activation, there is reason to hypothesize that adjunctive use of PD1/PD-L1 inhibitors could prove useful in improving efficacy of iNKTs responses against NB. In addition to the data on adoptive transfer of iNKTs in NB, iNKT adoptive transfer has been shown to reduce liver metastases of melanoma in a mouse model and has also demonstrated disease responses in patients with HNSCC (67, 68). Taken together, these pre-clinical NB studies and clinical studies in other solid tumor patients suggest that the adoptive cell transfer of iNKTs may offer a therapeutic and complementary role in NB by targeting TAMs and enhancing or restoring NK- and T-cell cytotoxicity. However, clinical trials of adoptive transfer of unmodified iNKTs have not yet been performed in patients with NB.

### CAR-iNKT Cells

CAR-modified iNKTs offer another area of great promise in the treatment of NB. GD2-specific CAR-iNKTs reduced the tumor volumes of xenografted CD1d– NB tumors in lymphocyte-deficient mice and prolonged survival (69). Additionally, in contrast to a comparison group in which these mice were treated with GD2-CAR T cells, CAR-iNKTs had significantly greater trafficking to NB tumors, and resulted in no graft vs. host disease (GVHD), while the CAR T cells showed liver and lung edema and lymphocytic infiltration consistent with GVHD (69). Although the reason for differences in GVHD between the CAR-iNKTs and CAR T cells is unknown, it is postulated that it may be due to the release of Th2-like cytokines by CD4+ CAR-iNKTs. Importantly, CAR-iNKTs retain both their ability to recognize CD1d/GAg complexes as well their cytotoxic activity against immunosuppressive TAMs (69). In a separate study, a subset of CAR-iNKTs that express CD62L were found to have five-fold longer persistence in host mice than CD62L- CAR-iNKTs (70). Artificial antigen presenting cells (aAPCs) were then created and used to enrich for CD62L+ iNKTs that were subsequently modified by CARs specific for GD2 and CD19 antigens. The CAR-iNKTs generated from CD62L+ enriched iNKTs were used in mice with NB and lymphoma, and demonstrated significantly longer *in vivo* persistence and therapeutic efficacy when compared with CAR-iNKTs generated without CD62L+ cell enrichment (70). These data provide an exciting new method for iNKT-CAR development that has not yet been tested clinically.

However, CAR-iNKTs are now being explored in a Phase I clinical trial (GINAKIT2 trial at Baylor) for patients with relapsed or refractory NB. This study aims to identify the maximum tolerated dose of CAR-iNKTs and involves the use of expanded autologous iNKTs modified with a GD2-CAR containing the IL-15 gene. This trial is currently recruiting and early results from two patients treated at the lowest dose level show that one patient's disease was stabilized, while the other had a significant partial response without dose-limiting toxicity (71). The iNKTs used in this clinical study are derived from expanded human peripheral blood mononuclear cells that have not been enriched using the aAPCs discussed above (70).

The potential advantages of CAR-iNKTs over CAR-Ts in NB include their relatively higher NB tumor penetration and reduced incidence of GVHD in pre-clinical models. Additionally, retention of the TCR function on CAR-iNKTs allows for clearance of immunosuppressive TAMs that contribute to tumor growth and refractoriness to immunotherapies. Given that GD2 CAR T cells have been limited due to decreased persistence and tumor penetration in NB, CAR-iNKTs offer an exciting potential solution for overcoming this barrier (10).

## Biology of NK Cells

### NK Activation and Activity in Neuroblastoma

NK cells are innate lymphoid cells that represent 5–15% of circulating lymphocytes and are important effectors against virus-infected, foreign, and cancerous cells. Human NKs are divided into a predominantly cytotoxic group found mainly in the peripheral blood (CD56^dim^CD16+) and a predominantly immunomodulatory group found mainly in lymph nodes (CD56^bright^CD16–) (72–74). The rapid response of NKs to their targets is mediated by a network of activating and inhibitory cell surface receptors that allow NKs to distinguish healthy “self” cells from cancerous, infected, or foreign cells (13). There are several groups of NK surface receptors including killer cell immunoglobin-like receptors (KIRs); the natural cytotoxicity receptors (NCRs) NKp30, NKp44, and NKp46; natural killer group 2D receptors (NKG2D); the Nectin family of receptors including DNAM-1 and CD96; the SLAM family; and FcγRIIIa (CD16). Cytokines also play a role in NK activation, and, as will be described below, are used clinically for NK-based therapies in NB. When activated, NKs utilize perforin, granzyme B, TRAIL (CD253), and FasL (CD178) to mediate cellular cytotoxicity. NKs also release cytokines and chemokines including IFN-γ, TNF-α, GM-CSF, MIP1-α, RANTES, IL-6, and CCL5 thus attracting and activating monocytes, macrophage, T cells, DCs, and neutrophils to promote an anti-tumor response (13, 75). We will first focus on the influence of NK surface proteins on outcomes in NB, and then discuss specific NK-based NB therapies.

### NK Receptors and Influence on Anti-tumor Activity

First, KIRs are a family of NK surface receptors that are largely inhibitory, and important in helping NKs to distinguish their targets from healthy cells. They are encoded by highly polymorphic germline genes, and recognize MHC-I as their ligand. When inhibitory KIRs are bound to matched MHC-I, they typically send inhibitory intracellular signals, thereby avoiding auto-reactivity. Importantly, when MHC-I is absent or downregulated (e.g., in cancerous, virus-infected, or transformed cells), NKs no longer receive inhibitory signaling from KIR-MHC-I binding, and are then positioned to become cytotoxic (13). Significantly, in NB, MHC-I downregulation is very common, resulting in a lack of KIR-mediated NK inhibition that allows NKs to recognize and kill these cancerous cells.

Interestingly, the type of KIRs expressed on NKs have been shown to affect clinical outcomes and predict response to immunotherapy with dinutuximab. A Children's Oncology Group (COG) study, ANBL0032, evaluated the influence of KIR and KIR-ligand genotype on the response of NB to immunotherapy. This study found that patients with genes for inhibitory KIR and KIR ligands (KIR-L) had the greatest improvement in EFS and OS when treated with dinutuximab, IL-2, and GM-CSF, and that these patients were responsible for most of the benefit from immunotherapy seen in the trial (76). In fact, those without inhibitory KIR/KIR-L genes did not have a significant difference in EFS or OS between the dinutuximab-containing arm and the non-dinutuximab-containing comparator arm. This counterintuitive finding suggests that perhaps the inhibitory KIR/KIR-L interaction that would have led to reduced NK anti-tumor cytotoxicity was mitigated by the immunostimulation with dinutuximab, IL-2, and GM-CSF. Supporting this hypothesis, in the group not treated with dinutuximab, those with inhibitory KIR/KIRL expression trended toward worsened survival compared with those without inhibitory KIR/KIRL expression, suggesting that immunotherapy with dinutuximab and cytokines IL-2 and GM-CSF may have restored the antitumor activity of otherwise inhibited NKs (76). Interestingly, this same effect was not seen in prior studies that used 3F8 (a murine-derived GD2-directed monoclonal antibody) but did not consistently utilize IL-2 (77–79). The difference in cytokine exposure between these studies may be relevant, as IL-2 does increase expression of activating NK receptors NKp30, NKp44, and DNAM-1 on NKs, potentially shifting the balance toward NK activation (80). The baseline expression of activating NK receptors has been shown to be relevant in NB as well. In another study that compared progression-free-survival of HRNB patients with greater expression of an activating NKp30 isoform, NKp30B, with patients expressing an inhibitory NKp30 isoform, NKp30C, it was found that those with greater NKp30B relative to NKp30C expression had improved progression-free survival (81).

**Figure 3:**
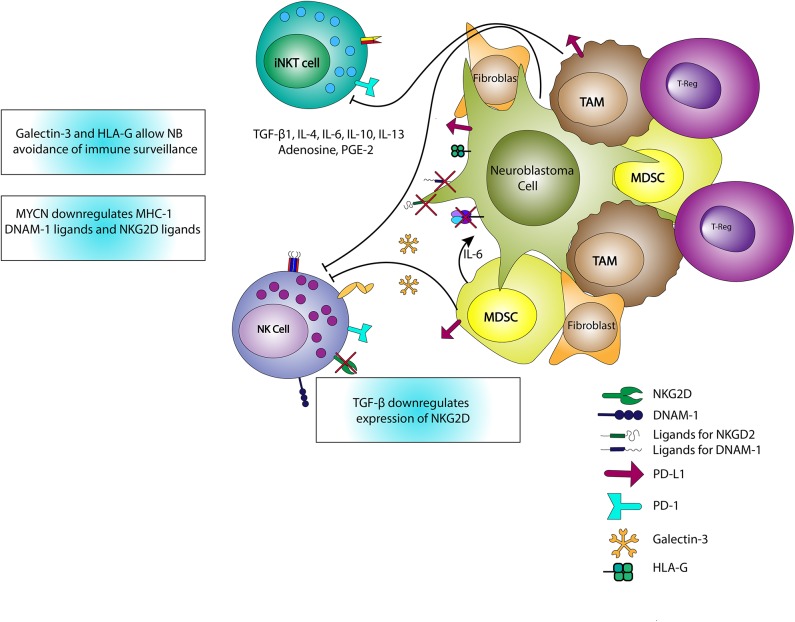
Interactions of iNKTs, NKs, and the TME. iNKTs and NKs are inhibited through the release of TGF-β, IL-4, IL-6, IL-10, IL-13, adenosine, and PGE-2. IL-6 also stimulates the growth of neuroblasts and increases osteoclastic activity associated with metastatic potential. TGF-β reduces expression of the activating NK receptor, NKG2D, limiting NK activation. MYCN expressed in neuroblasts downregulates MHC-I expression and ligands for NK-activating receptors including DNAM-1 and NKG2D ligands, thereby promoting immune evasion. Galectin-3, HLA-G, and PD-L1 expression also contribute to immune evasion or downregulation in the TME.

Another important factor in NK activation in the NB TME is the presence of NK-activating ligands on the NB cell surface (Figure 3). As previously mentioned, neuroblasts downregulate MHC-I allowing for NK recognition and activation. However, in NB, NKs also recognize activation signals from proteins on neuroblasts including polio-virus receptor (PVR) and NCR-ligands via their DNAM-1 and NCRs (82). Additionally, cancer stem cells (CSCs), which are often refractory to radiation and chemotherapy, express stress ligands recognizable by NKG2D with resultant NK-mediated cytotoxicity (83). Thus, for NKs in NB, the combination of downregulated MHC-I proteins and expression of NK receptor ligands provides an environment conducive to NK activation and killing of neuroblasts and CSCs. Furthermore, the influence of NK receptors on NB outcomes demonstrates the potential of NKs as therapeutic tools, and also represent an area for personalized medicine given the differential response to immunotherapies in the case of dinutuximab. The influence of NK receptors on clinical outcome has previously been shown in other cancer types as well (84–87).

### Neuroblastoma TME and NK Cells

While there are numerous advantageous features of NKs in the NB TME, in this environment NKs can also be downregulated by T_REG_, MDSCs, TAMs, immature DCs, and neuroblasts (88–90). *MYCN*, a gene whose amplification is associated with HRNB, downregulates NB expression of ligands for NKG2D and DNAM-1, resulting in a reduction in NK recognition and cytotoxicity (91). TGF-β1 release decreases NK expression of activating receptors including NKG2D, NKp44, and NKp30 (80, 92). Additionally, NKs are inhibited through cell-cell interactions with tumor-associated fibroblasts in the presence of PGE-2 (80). Notably, these tumor-associated fibroblasts are also associated with increased NB cell proliferation and Schwannian stroma-poor tumors (93, 94). Neuroblasts themselves express B7-H3 which binds to an inhibitory NK receptor, limiting NK killing (82). NKs counteract the immunosuppressive solid TME via cytokine release), activation and/or maturation of other immune cells, and release of exosomal microRNAs (miRs). For example, miR-186 limits NB expression of MYCN, AURKA, TGF-βR1 and TGF-βR2, to reduce TGF-β-mediated immune escape, restrict tumorigenesis, and enhance NB cytotoxicity (95).

Taken together, NKs are associated with an improved prognosis in NB and have advantageous features for use in NB immunotherapy. Since particular NK KIR genotypes and activating cell surface receptor expression patterns appear to confer improved prognosis and predict response to immunotherapy treatment, it will be vital to identify treatment options that favor activation and avoid inhibition triggered by known NK surface receptor pathways. We will now review available data regarding the use of NK-based therapies in NB.

## NK Cells in the Treatment of Neuroblastoma

### Soluble Factors Activating NK Cells in Neuroblastoma

**Figure 4:**
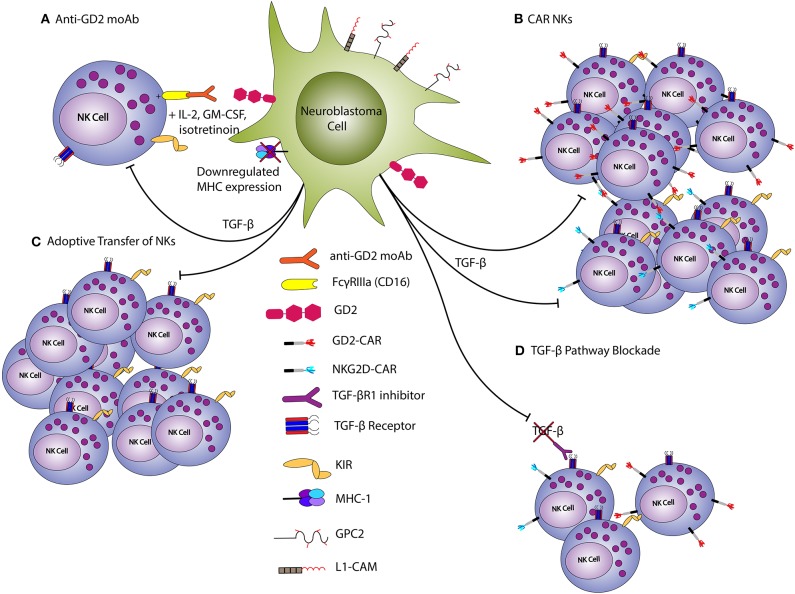
NK-based treatments of neuroblastoma. Endogenous NKs can be activated against NB via anti-GD2 monoclonal antibodies **(A)** Conversely, CAR-modified NKs **(B)** or those expanded/stimulated with cytokines **(C)** can be adoptively transferred to disease bearing hosts. TGF-β inhibits NK anti-tumor function, and blockade helps to promote an anti-tumor response **(D)**. TGF-β blockade can thus be used to reduce the inhibition of endogenous or adoptively-transferred NKs. The downregulation of MHC expression on neuroblasts allows for NK activation, as inhibitory killer immunoglobulin receptors (KIRs) are not ligated.

The most prominent example of immunotherapy for HRNB is dinutuximab, the first ever FDA-approved immunotherapy for NB (6) (Figure 4). Dinutuximab is a monoclonal antibody specific for GD2, and is believed to exert its effects at least in part via the binding of its Fc domain to NK Fc receptors (FcγRIIIa; CD16). Indeed, combination therapy with dinutuximab, IL-2, and GM-CSF resulted in augmented activation of NKs and led to increased ADCC of GD2-expressing NB cells (96). The use of anti-GD2 antibody in HRNB improved 2-year event-free-survival and this therapy is now standard of care for HRNB patients (6). In the setting of relapsed/refractory disease, dinutuximab used with irinotecan and temozolomide resulted in disease response in >50% vs. 6% in a comparator arm with irinotecan and temozolomide, but without dinutuximab (7). Another method of activating NKs is the development of bi- and tri-specific killer cell engagers (BiKEs and TriKEs, respectively), which promote the simultaneous engagement of tumor associated antigens (TAAs) and activating receptors on NKs (97–99). Several BiKEs and TriKEs have been created using CD16 or NKG2D as the primary mediators to increase NK cytotoxicity in *in vitro* and *in vivo* models of multiple myeloma, Hodgkin lymphoma and AML (100–102). Recently, Gauthier et al. showed that TriKEs targeting both CD16 and NKp46, and a TAA showed augmented killing potency in both *in vitro* and *in vivo* models of solid and liquid tumors when compared to monoclonal antibodies against TAAs (103). However, the role of BiKEs and TriKEs in NB is yet to be delineated.

### NK Adoptive Cellular Therapy

Adoptive NK therapy involves obtaining autologous or allogeneic peripheral blood or bone marrow-derived NKs and re-infusing these cells into a patient to mediate antitumor effects. Despite promising *in vitro* results for autologous NK therapy, only limited antitumor efficacy was observed in patients with metastatic solid tumors (104, 105). The lack of success in autologous NK therapy is thought to be due to a lack of KIR-mismatch. On the other hand, allogeneic adoptive transfer of NKs has demonstrated promising cytotoxic activity against several malignancies (106). In allogeneic adoptive NK transfer, peripheral blood or bone marrow of an HLA-matched or haploidentical donor is collected, depleted of T cells, and NKs are isolated, expanded and activated before adoptive transfer to a patient. In one study of adoptive transfer of allogeneic NKs in AML patients, infusion of KIR-HLA-mismatched NKs to 10 AML patients with minimal residual disease (MRD) negative status, resulted in 100% EFS at 2 years (107). Additionally, in adult AML patients receiving allogeneic SCT, improved prognosis was observed when there was KIR-HLA mismatch (84, 86, 87).

In a mouse model of NB, transfer of activated NKs in combination with anti-GD2 antibody led to an increase in survival (108). Moreover, the combination of a TGF-βR1 inhibitor (galunisertib), NK allogeneic infusion, and dinutuximab showed enhanced NK-mediated cytotoxicity in mice xenografted with NB cell lines or patient-derived NB (109). This effect was mediated by decreasing the immunosuppressive effect of TGF-β1 on NKs, which reversed the decreased expression of TRAIL, DNAM-1, NKG2D, and NKp30 (109). Similar strategies have been employed in clinical trials (7). In one study, 35 patients with refractory or progressive HRNB were treated with allogeneic NKs and 3F8, a murine-derived antibody with higher binding affinity for GD2 than dinutuximab; this treatment resulted in an overall response in 28% of patients, stable disease in 49%, and progressive disease in 23% (110). The same group is currently conducting a clinical trial, NCT 02650648, evaluating the use of allogeneic NKs with cyclophosphamide conditioning, humanized 3F8 antibody, and IL-2 administration. A separate trial used parental NK infusions (with maximal KIR-recipient KIR-ligand mismatches), in combination with humanized ch14.18K322A, an anti-GD2 antibody with the same binding affinity as dinutuximab, along with GM-CSF, IL-2, and various chemotherapeutic agents in 13 patients with HRNB. This study demonstrated impressive results with 61.5% (8 out of 13 patients) having a complete or partial response, and 38.5% (5 out of 13 patients) having a stable disease (111). Finally, another ongoing trial, NCT03209869, is employing four cycles of chemotherapy followed by adoptive transfer of haploidentical NKs grown *ex vivo*, and subsequent administration of anti-GD2 antibody fused with IL-2. Results from these ongoing clinical trials should be very illuminating (Table 1).

**Table 1:** Ongoing clinical trials using NK cell-based therapies for neuroblastoma.

**Title**	**Status**	**Phase**	**NCT number**	**Sponsor**	**Study start date**	**Anticipated completion date**
Haploidentical stem cell transplantation and NK cell therapy in patients with high-risk solid tumors	Active, not recruiting	2	01807486	Samsung medical center	05/2013	6/2019
Phase II STIR trial: haploidentical transplant and donor NK cells for solid tumors	Recruiting	2	02100891	Monica thakar medical college of wisconsin	03/2014	12/2021
Immunotherapy of neuroblastoma patients using a combination of anti-GD2 and NK cells	Recruiting	1,2	03242603	National university hospital, singapore	10/2017	8/2020
NK cell infusions with irinotecan, temozolomide, and dinutuximab	Not yet recruiting	1,2	04211675	Nationwide children's hospital	4/2020	12/2023
Immunotherapy of relapsed refractory neuroblastoma with expanded NK cells	Recruiting	1	02573896	New approaches to neuroblastoma therapy consortium	11/2018	8/2022
Humanized anti-GD2 antibody Hu3F8 and allogeneic NK cells for high-risk neuroblastoma	Recruiting	1	02650648	Memorial sloan kettering cancer center	01/2016	1/2021
Treatment of relapsed or refractory neuroblastoma with expanded haploidentical NK cells and Hu14.18-IL2	Recruiting	1	03209869	University of wisconsin	3/2018	9/2021

### CAR-NK Cells

Another promising NB treatment approach involves CAR-modified NKs, which can be manufactured using NKs derived from peripheral blood, umbilical cord blood, bone marrow, embryonic stem cells, induced pluripotent stem cells, or NK lines (NK-92). CAR-modified NKs retain their ability to recognize tumor cells that have down-regulated MHC-I, and induce a more muted cytokine release syndrome (CRS) and GVHD than CAR T cells (112–114). Since the initial report of a CAR-NK in 1995, in which retrovirally transduced human NKs were made to express CD4ζ, several other CAR-NKs have been generated (115, 116). These CAR-NKs target a variety of TAAs including CD19, CD20, CD7, CD33, MUC-1, mesothelin, and HER-2, and demonstrate the ability to kill TAA-expressing targets. In a recent study, induced pluripotent stem cell-derived NKs transformed with mesothelin-directed CARs containing NK-specific activation domains from NKG2D, 2B4, and the CD3ζ domain, showed improved *in vivo* cytotoxicity, improved mouse survival, and fewer adverse events than CAR T cells in an ovarian cancer xenograft model (117). Additionally, the NKs transduced with the CAR containing NKG2D, 2B4, and the CD3ζ activation domains had greater cytotoxicity and IFN-γ release than NKs transduced with CARs comprised of CD28, 4-1BB, and CD3 ζ, which are more commonly employed for CAR T cell manufacturing (117). In the clinical domain, IL-15-expressing, CD19-directed CAR-NKs bearing an inducible caspase 9 suicide gene were created using retroviral transduction of NKs derived from human cord blood. These CD19-directed CAR-NKs were used to treat 11 patients with non-Hodgkin lymphoma or chronic lymphocytic leukemia in a Phase I/II clinical trial, with 7 out of 11 (64%) achieving a complete remission (116).

Various CAR-NKs have also been tested in NB. In one study, NK-92 cells were transduced with a CAR containing an anti-GD2 scFv along with CD3ζ domains; these GD2-CAR NKs demonstrated robust specific cytotoxicity against GD2+ neuroblasts and other GD2-expressing tumor cells (118). In another report, NKs were transduced with a CAR containing NKG2D, DAP10, and CD3ζ domains, and when used to against leukemia and sarcoma cell lines, mediated substantial tumor cytotoxicity (119). NKG2D CAR-NKs were also shown to be capable of cytotoxicity against MDSCs in the NB TME, which express high levels of NKG2D ligands (120). The elimination of MDSCs then allowed for increased infiltration and tumor cytotoxicity by GD2 CAR T cells infused after administration of the CAR-NKs (120). These observations illustrate the potential for complementary approaches using CAR-NKs and CAR T cells for solid tumor immunotherapy.

## Challenges for NK Cell-Based Therapies

Despite the promise of NK-based therapies, several challenges still exist. These include limited *in vivo* proliferation and persistence of CAR NKs *in vivo*, and the immunosuppressive TME of solid tumors. Use of different cytokines such as IL-15 and IL-21 have been shown to result in better antitumor activity of NKs while avoiding the simultaneous survival and expansion of T_REG_ that has been seen with the use of IL-2 (113, 121, 122). Moreover, as discussed above, the TME of solid tumors can express appreciable levels of TGFβ1 as well as checkpoint ligands (e.g., PD-L1); as such, the use of TGFβR1 or PD-1 inhibitors may enhance NK efficacy (123–125). In addition, several obstacles need to be overcome to allow for the use of CAR-NKs in clinical practice. The optimal composition of CAR-NKs has not been identified yet; focus on this will result in CARs that promote improved effector function and persistence (102). In conclusion, future studies to refine and optimize CAR-NK manufacturing may allow for translation of the promising *in vitro* and *in vivo* results into clinically improved outcomes for NB patients, as such optimization and experimental refinement have done for CAR-T therapeutics.

## Conclusion

iNKTs and NKs have unique and complementary features that hold promise in the treatment of NB and other solid tumors. In particular, the abilities of NKs and iNKTs to kill TAMs and MDSCs, mature dendritic cells, and robustly release proinflammatory cytokines to recruit and activate conventional T cells, make these cells powerful tools in the armamentarium for NB therapy. The ability of these cells to break down the barriers that have previously limited CAR T cell therapies in solid tumors (limited T cell persistence and potency, inability to kill tumors without target TAA or MHC-I expression, and an immunosuppressive TME), and promising pre-clinical data suggest great potential for iNKT and NK therapies in NB. The potential for therapeutic synergy with CAR T cells and checkpoint inhibitors is an exciting area of future study.

## Author Contributions

KM wrote the initial draft with initial edits and contributions by SK. KM prepared the figures with editorial input from HB. MH edited and oversaw focus of article and provided critical revisions. HB provided oversight of article construction and edited all interim drafts with critical revisions. All authors participated in the editing of the final document.

## Conflict of Interest

The authors declare that the research was conducted in the absence of any commercial or financial relationships that could be construed as a potential conflict of interest.

## References

[1] Coughlan D Gianferante M Lynch CF Stevens JL Harlan LC. Treatment and survival of childhood neuroblastoma: evidence from a population-based study in the United States. Pediatr Hematol Oncol. (2017) 34:320–30. 10.1080/08880018.2017.1373315 29039999 PMC6764456

[2] Cohn SL Pearson AD London WB Monclair T Ambros PF Brodeur GM. The international neuroblastoma risk group (INRG) classification system: an INRG task force report. J Clin Oncol. (2009) 27:289–97. 10.1200/JCO.2008.16.6785 19047291 PMC2650388

[3] Friedman DN Henderson TO. Late effects and survivorship issues in patients with neuroblastoma. Children. (2018) 5:107. 10.3390/children5080107 30082653 PMC6111874

[4] Laverdiere C Cheung NK Kushner BH Kramer K Modak S LaQuaglia MP. Long-term complications in survivors of advanced stage neuroblastoma. Pediatr Blood Cancer. (2005) 45:324–32. 10.1002/pbc.20331 15714447

[5] Portwine C Rae C Davis J Teira P Schechter T Lewis V. Health-related quality of life in survivors of high-risk neuroblastoma after stem cell transplant: a national population-based perspective. Pediatr Blood Cancer. (2016) 63:1615–21. 10.1002/pbc.26063 27203368

[6] Yu AL Gilman AL Ozkaynak MF London WB Kreissman SG Chen HX Children's Oncology, anti-GD2 antibody with GM-CSF, interleukin-2, and isotretinoin for neuroblastoma. N Engl J Med. (2010) 363:1324–34. 10.1056/NEJMoa0911123 20879881 PMC3086629

[7] Mody R Naranjo A Van Ryn C Yu AL London WB Shulkin BL. Irinotecan-temozolomide with temsirolimus or dinutuximab in children with refractory or relapsed neuroblastoma (COG ANBL1221): an open-label, randomised, phase 2 trial. Lancet Oncol. (2017) 18:946–57. 10.1016/S1470-2045(17)30355-8 28549783 PMC5527694

[8] Maude SL Laetsch TW Buechner J Rives S Boyer M Bittencourt H. Tisagenlecleucel in children and young adults with B-cell lymphoblastic leukemia. N Engl J Med. (2018) 378:439–48. 10.1056/NEJMoa1709866 29385370 PMC5996391

[9] Merchant MS Wright M Baird K Wexler LH Rodriguez-Galindo C Bernstein D. Phase I clinical trial of ipilimumab in pediatric patients with advanced solid tumors. Clin Cancer Res. (2016) 22:1364–70. 10.1158/1078-0432.CCR-15-0491 26534966 PMC5027962

[10] Richards RM Sotillo E Majzner RG. CAR T cell therapy for neuroblastoma. Front Immunol. (2018) 9:2380. 10.3389/fimmu.2018.02380 30459759 PMC6232778

[11] Lampson LA Whelan JP Fisher CA. HLA-A,B,C and beta 2-microglobulin are expressed weakly by human cells of neuronal origin, but can be induced in neuroblastoma cell lines by interferon. Prog Clin Biol Res. (1985) 175:379–88. 3921986

[12] Wolf BJ Choi JE Exley MA. Novel approaches to exploiting invariant NKT cells in cancer immunotherapy. Front Immunol. (2018) 9:384. 10.3389/fimmu.2018.00384 29559971 PMC5845557

[13] Guillerey C Huntington ND Smyth MJ. Targeting natural killer cells in cancer immunotherapy. Nat Immunol. (2016) 17:1025–36. 10.1038/ni.3518 27540992

[14] Hishiki T Mise N Harada K Ihara F Takami M Saito T. Frequency and proliferative response of circulating invariant natural killer T cells in pediatric patients with malignant solid tumors. Pediatr Surg Int. (2018) 34:169–76. 10.1007/s00383-017-4185-1 29018953

[15] Krasnova Y Putz EM Smyth MJ Souza-Fonseca-Guimaraes F. Bench to bedside: NK cells and control of metastasis. Clin Immunol. (2017) 177:50–9. 10.1016/j.clim.2015.10.001 26476139

[16] Kmieciak M Basu D Payne KK Toor A Yacoub A Wang XY. Activated NKT cells and NK cells render T cells resistant to myeloid-derived suppressor cells and result in an effective adoptive cellular therapy against breast cancer in the FVBN202 transgenic mouse. J Immunol. (2011) 187:708–17. 10.4049/jimmunol.1100502 21670315 PMC3131490

[17] Bassiri H Das R Nichols KE. Invariant NKT cells: killers and conspirators against cancer. Oncoimmunology. (2013) 2:e27440. 10.4161/onci.27440 24575380 PMC3926875

[18] Crowe NY Smyth MJ Godfrey DI. A critical role for natural killer T cells in immunosurveillance of methylcholanthrene-induced sarcomas. J Exp Med. (2002) 196:119–27. 10.1084/jem.20020092 12093876 PMC2194015

[19] Cui J Shin T Kawano T Sato H Kondo E Toura I. Requirement for Valpha14 NKT cells in IL-12-mediated rejection of tumors. Science. (1997) 278:1623–6. 10.1126/science.278.5343.1623 9374462

[20] Molling JW Langius JA Langendijk JA Leemans CR Bontkes HJ van der Vliet HJ. Low levels of circulating invariant natural killer T cells predict poor clinical outcome in patients with head and neck squamous cell carcinoma. J Clin Oncol. (2007) 25:862–8. 10.1200/JCO.2006.08.5787 17327607

[21] Exley MA Lynch L Varghese B Nowak M Alatrakchi N Balk SP. Developing understanding of the roles of CD1d-restricted T cell subsets in cancer: reversing tumor-induced defects. Clin Immunol. (2011) 140:184–95. 10.1016/j.clim.2011.04.017 21646050 PMC3143311

[22] Dhodapkar MV Geller MD Chang DH Shimizu K Fujii S Dhodapkar KM. A reversible defect in natural killer T cell function characterizes the progression of premalignant to malignant multiple myeloma. J Exp Med. (2003) 197:1667–76. 10.1084/jem.20021650 12796469 PMC2193955

[23] Fujii S Shimizu K Klimek V Geller MD Nimer SD Dhodapkar MV. Severe and selective deficiency of interferon-gamma-producing invariant natural killer T cells in patients with myelodysplastic syndromes. Br J Haematol. (2003) 122:617–22. 10.1046/j.1365-2141.2003.04465.x 12899717

[24] Tahir SM Cheng O Shaulov A Koezuka Y Bubley GJ Wilson SB. Loss of IFN-gamma production by invariant NK T cells in advanced cancer. J Immunol. (2001) 167:4046–50. 10.4049/jimmunol.167.7.4046 11564825

[25] Berzins SP Smyth MJ Baxter AG. Presumed guilty: natural killer T cell defects and human disease. Nat Rev Immunol. (2011) 11:131–42. 10.1038/nri2904 21267014

[26] Courtney AN Tian GW Marinova E Wei J Guo LJ Jin JL NKT cells control tumor associated macrophages and metastatic growth in neuroblastoma. J Immunol. (2017) 198:24 Available online at: https://www.jimmunol.org/content/198/1_Supplement/204.24

[27] Swann JB Uldrich AP van Dommelen S Sharkey J Murray WK Godfrey DI. (2009). Type I natural killer T cells suppress tumors caused by p53 loss in mice. Blood. 113:6382–5. 10.1182/blood-2009-01-198564 19234138 PMC2710930

[28] Hishiki T Mise N Harada K Ihara F Takami M Saito T. Invariant natural killer T infiltration in neuroblastoma with favorable outcome. Pediatr Surg Int. (2018) 34:195–201. 10.1007/s00383-017-4189-x 29018959

[29] Bedard M Salio M Cerundolo V. Harnessing the power of invariant natural killer T cells in cancer immunotherapy. Front Immunol. (2017) 8:1829. 10.3389/fimmu.2017.01829 29326711 PMC5741693

[30] Brennan PJ Tatituri RV Brigl M Kim EY Tuli A Sanderson JP. Invariant natural killer T cells recognize lipid self antigen induced by microbial danger signals. Nat Immunol. (2011) 12:1202–11. 10.1038/ni.2143 22037601 PMC3242449

[31] Torreno-Pina JA Manzo C Salio M Aichinger MC Oddone A Lakadamyali M. The actin cytoskeleton modulates the activation of iNKT cells by segregating CD1d nanoclusters on antigen-presenting cells. Proc Natl Acad Sci USA. (2016) 113:E772–81. 10.1073/pnas.1514530113 26798067 PMC4760795

[32] Reilly EC Wands JR Brossay L. Cytokine dependent and independent iNKT cell activation. Cytokine. (2010) 51:227–31. 10.1016/j.cyto.2010.04.016 20554220 PMC2914806

[33] Tyznik AJ Tupin E Nagarajan NA Her MJ Benedict CA Kronenberg M. Cutting edge: the mechanism of invariant NKT cell responses to viral danger signals. J Immunol. (2008) 181:4452–6. 10.4049/jimmunol.181.7.4452 18802047 PMC2597678

[34] Wesley JD Tessmer MS Chaukos D Brossay L. NK cell-like behavior of Valpha14i NK T cells during MCMV infection. PLoS Pathog. (2008) 4:e1000106. 10.1371/journal.ppat.1000106 18636102 PMC2442879

[35] Vinay DS Choi BK Bae JS Kim WY Gebhardt BM Kwon BS. CD137-deficient mice have reduced NK/NKT cell numbers and function, are resistant to lipopolysaccharide-induced shock syndromes, and have lower IL-4 responses. J Immunol. (2008) 173:4218–29. 10.4049/jimmunol.173.6.4218 15356173

[36] Wang J Cheng L Wondimu Z Swain M Santamaria P Yang Y. Cutting edge: CD28 engagement releases antigen-activated invariant NKT cells from the inhibitory effects of PD-1. J Immunol. (2009) 182:6644–7. 10.4049/jimmunol.0804050 19454657

[37] Wang XF Lei Y Chen M Chen CB Ren H Shi TD. PD-1/PDL1 and CD28/CD80 pathways modulate natural killer T cell function to inhibit hepatitis B virus replication. J Viral Hepat. (2013) 20(Suppl. 1):27–39. 10.1111/jvh.12061 23458522

[38] Kaneda H Takeda K Ota T Kaduka Y Akiba H Ikarashi Y. ICOS costimulates invariant NKT cell activation. Biochem Biophys Res Commun. (2005) 327:201–7. 10.1016/j.bbrc.2004.12.004 15629449

[39] Kuylenstierna C Bjorkstrom NK Andersson SK Sahlstrom P Bosnjak L Paquin-Proulx D NKG2D performs two functions in invariant NKT cells: direct TCR-independent activation of NK-like cytolysis and co-stimulation of activation by CD1d. Eur J Immunol. (2011) 411:913–23. 10.1002/eji.200940278 PMC3523190 21590763

[40] King LA Lameris R de Gruijl TD van der Vliet HJ CD1d-invariant natural killer T cell-based cancer immunotherapy: alpha-galactosylceramide and beyond. Front Immunol. (2018) 9:1519 10.3389/fimmu.2018.02606 30013569 PMC6036112

[41] Kitamura H Iwakabe K Yahata T Nishimura S Ohta A Ohmi Y. The natural killer T (NKT) cell ligand alpha-galactosylceramide demonstrates its immunopotentiating effect by inducing interleukin (IL)-12 production by dendritic cells and IL-12 receptor expression on NKT cells. J Exp Med. (1999) 189:1121–8. 10.1084/jem.189.7.1121 10190903 PMC2193012

[42] Nair S Dhodapkar MV. Natural killer T cells in cancer immunotherapy. Front Immunol. (2017) 8:1178. 10.3389/fimmu.2017.01178 29018445 PMC5614937

[43] Hung JT Huang JR Yu AL. Tailored design of NKT-stimulatory glycolipids for polarization of immune responses. J Biomed Sci. (2017) 24:22. 10.1186/s12929-017-0325-0 28335781 PMC5364570

[44] Fujio M Wu D Garcia-Navarro R Ho DD Tsuji M Wong CH. Structure-based discovery of glycolipids for CD1d-mediated NKT cell activation: tuning the adjuvant versus immunosuppression activity. J Am Chem Soc. (2006) 128:9022–3. 10.1021/ja062740z 16834361

[45] Huang JR Tsai YC Chang YJ Wu JC Hung JT Lin KH. alpha-galactosylceramide but not phenyl-glycolipids induced NKT cell anergy and IL-33-mediated myeloid-derived suppressor cell accumulation via upregulation of egr2/3. J Immunol. (2014) 192:1972–81. 10.4049/jimmunol.1302623 24465013

[46] Parekh VV. Glycolipid antigen induces long-term natural killer T cell anergy in mice. J Clin Invest. (2005) 115:2572–83. 10.1172/JCI24762 16138194 PMC1193878

[47] Metelitsa LS. Anti-tumor potential of type-I NKT cells against CD1d-positive and CD1d-negative tumors in humans. Clin Immunol. (2011) 140:119–29. 10.1016/j.clim.2010.10.005 21095162 PMC3444285

[48] Song L Asgharzadeh S Salo J Engell K Wu HW Sposto R. Valpha24-invariant NKT cells mediate antitumor activity via killing of tumor-associated macrophages. J Clin Invest. (2009) 119:1524–36. 10.1172/JCI37869 19411762 PMC2689106

[49] Sohara Y Shimada H Minkin C Erdreich-Epstein A Nolta JA DeClerck YA. Bone marrow mesenchymal stem cells provide an alternate pathway of osteoclast activation and bone destruction by cancer cells. Cancer Res. (2005) 65:1129–35. 10.1158/0008-5472.CAN-04-2853 15734993

[50] Liu D Song L Wei J Courtney AN Gao X Marinova E. IL-15 protects NKT cells from inhibition by tumor-associated macrophages and enhances antimetastatic activity. J Clin Invest. (2012) 122:2221–33. 10.1172/JCI59535 22565311 PMC3366399

[51] Mussai F Carmela DS Cerundolo V. Interaction between invariant NKT cells and myeloid-derived suppressor cells in cancer patients. J Immunother. (2012) 35:449–59. 10.1097/CJI.0b013e31825be926 22735803

[52] Beldi-Ferchiou A Caillat-Zucman S. Control of NK cell activation by immune checkpoint molecules. Int J Mol Sci. (2017) 18:2129. 10.3390/ijms18102129 29023417 PMC5666811

[53] Bellucci R Martin A Bommarito D Wang K Hansen SH Freeman GJ. Interferon-gamma-induced activation of JAK1 and JAK2 suppresses tumor cell susceptibility to NK cells through upregulation of PD-L1 expression. Oncoimmunology. (2015) 4:e1008824. 10.1080/2162402X.2015.1008824 26155422 PMC4485824

[54] Chang WS Kim JY Kim YJ Kim YS Lee JM Azuma M. Cutting edge: programmed death-1/programmed death ligand 1 interaction regulates the induction and maintenance of invariant NKT cell anergy. J Immunol. (2015) 181:6707–10. 10.4049/jimmunol.181.10.6707 18981087

[55] Hsu J Hodgins JJ Marathe M Nicolai CJ Bourgeois-Daigneault MC Trevino TN. Contribution of NK cells to immunotherapy mediated by PD-1/PD-L1 blockade. J Clin Invest. (2018) 128:4654–68. 10.1172/JCI99317 30198904 PMC6159991

[56] Parekh VV Lalani S Kim S Halder R Azuma M Yagita H. PD-1/PD-L blockade prevents anergy induction and enhances the anti-tumor activities of glycolipid-activated invariant NKT cells. J Immunol. (2009) 182:2816–26. 10.4049/jimmunol.0803648 19234176 PMC2709814

[57] Durgan K Ali M Warner P Latchman YE. Targeting NKT cells and PD-L1 pathway results in augmented anti-tumor responses in a melanoma model. Cancer Immunol Immunother CII. (2011) 60:547–58. 10.1007/s00262-010-0963-5 21240487 PMC3207499

[58] Guo Y Feng X Jiang Y Shi X Xing X Liu X. PD1 blockade enhances cytotoxicity of *in vitro* expanded natural killer cells towards myeloma cells. Oncotarget. (2016) 7:48360–74. 10.18632/oncotarget.10235 27356741 PMC5217023

[59] Giaccone G Punt CJ Ando Y Ruijter R Nishi N Peters M. A phase I study of the natural killer T-cell ligand alpha-galactosylceramide (KRN7000) in patients with solid tumors. Clin Cancer Res. (2002) 8:3702–9. Available online at: https://clincancerres.aacrjournals.org/content/8/12/3702.long 12473579

[60] Ishikawa A Motohashi S Ishikawa E Fuchida H Higashino K Otsuji M. A phase I study of alpha-galactosylceramide (KRN7000)-pulsed dendritic cells in patients with advanced and recurrent non-small cell lung cancer. Clin Cancer Res. (2005) 11:1910–7. 10.1158/1078-0432.CCR-04-1453 15756017

[61] Nagato K Motohashi S Ishibashi F Okita K Yamasaki K Moriya Y. Accumulation of activated invariant natural killer T cells in the tumor microenvironment after alpha-galactosylceramide-pulsed antigen presenting cells. J Clin Immunol. (2012) 32:1071–81. 10.1007/s10875-012-9697-9 22534863

[62] Nieda M Okai M Tazbirkova A Lin H Yamaura A Ide K. Therapeutic activation of Valpha24+Vbeta11+ NKT cells in human subjects results in highly coordinated secondary activation of acquired and innate immunity. Blood. (2004) 103:383–9. 10.1182/blood-2003-04-1155 14512316

[63] Metelitsa LS Naidenko OV Kant A Wu HW Loza MJ Perussia B. Human NKT cells mediate antitumor cytotoxicity directly by recognizing target cell CD1d with bound ligand or indirectly by producing IL-2 to activate NK cells. J Immunol. (2001) 167:3114–22. 10.4049/jimmunol.167.6.3114 11544296

[64] Mise N Takami M Suzuki A Kamata T Harada K Hishiki T. Antibody-dependent cellular cytotoxicity toward neuroblastoma enhanced by activated invariant natural killer T cells. Cancer Sci. (2016) 107:233–41. 10.1111/cas.12882 26749374 PMC4814252

[65] Bae EA Seo H Kim BS Choi J Jeon I Shin KS. Activation of NKT cells in an anti-PD-1-resistant tumor model enhances antitumor immunity by reinvigorating exhausted CD8 T cells. Cancer Res. (2018) 78:5315–26. 10.1158/0008-5472.CAN-18-0734 30012672

[66] Courtney AN Cross-talk between NKT cells and tumor associated macrophages in the tumor microenvironment. J Immunol. (2016) 196:7 Available online at: https://www.jimmunol.org/content/196/1_Supplement/142.7s

[67] Kunii N Horiguchi S Motohashi S Yamamoto H Ueno N Yamamoto S. Combination therapy of *in vitro*-expanded natural killer T cells and alpha-galactosylceramide-pulsed antigen-presenting cells in patients with recurrent head and neck carcinoma. Cancer Sci. (2009) 100:1092–8. 10.1111/j.1349-7006.2009.01135.x 19302288 PMC11158111

[68] Shin T Nakayama T Akutsu Y Motohashi S Shibata Y Harada M. Inhibition of tumor metastasis by adoptive transfer of IL-12-activated Valpha14 NKT cells. Int J Cancer. (2001) 91:523–8. 10.1002/1097-0215(20010215)91:4<523::AID-IJC1087>3.0.CO;2-L 11251976

[69] Heczey A Liu D Tian G Courtney AN Wei J Marinova E. Invariant NKT cells with chimeric antigen receptor provide a novel platform for safe and effective cancer immunotherapy. Blood. (2014) 124:2824–33. 10.1182/blood-2013-11-541235 25049283 PMC4215313

[70] Tian G Courtney AN Jena B Heczey A Liu D Marinova E. CD62L+ NKT cells have prolonged persistence and antitumor activity *in vivo*. J Clin Invest. (2016) 126:2341–55. 10.1172/JCI83476 27183388 PMC4887157

[71] Nowers CSCBKWT Cell Medica Collaborators, Baylor College of Medicine and Texas Children's Hospital, Present Positive Early Patient Data From CAR-NKT Neuroblastoma Trial at American Society Of Gene & Cell Therapy 22nd Annual Meeting. Washington, DC (2019).

[72] Abel AM Yang C Thakar MS Malarkannan S. Natural killer cells: development, maturation, and clinical utilization. Front Immunol. (2018) 9:1869. 10.3389/fimmu.2018.01869 30150991 PMC6099181

[73] Cantoni C Huergo-Zapico L Parodi M Pedrazzi M Mingari MC Moretta A. NK cells, tumor cell transition, and tumor progression in solid malignancies: new hints for NK-based immunotherapy?. J Immunol Res. (2016) 2016:4684268. 10.1155/2016/4684268 27294158 PMC4880686

[74] Cooper MA Fehniger TA Caligiuri MA. The biology of human natural killer-cell subsets. Trends Immunol. (2001) 22:633–40. 10.1016/S1471-4906(01)02060-9 11698225

[75] Sungur CM Murphy WJ. Positive and negative regulation by NK cells in cancer. Crit Rev Oncog. (2014) 19:57–66. 10.1615/CritRevOncog.2014010805 24941373 PMC4242411

[76] Erbe AK Wang W Carmichael L Kim K Mendonca EA Song Y. (2018). Neuroblastoma patients' KIR and KIR-ligand genotypes influence clinical outcome for dinutuximab-based immunotherapy: a report from the children's oncology group. Clin Cancer Res. (2016) 24:189–96. 10.1158/1078-0432.CCR-17-1767 28972044 PMC5754221

[77] Cheung NK Cheung IY Kushner BH Ostrovnaya I Chamberlain E Kramer K. Murine anti-GD2 monoclonal antibody 3F8 combined with granulocyte-macrophage colony-stimulating factor and 13-cis-retinoic acid in high-risk patients with stage 4 neuroblastoma in first remission. J Clin Oncol. (2012) 30:3264–70. 10.1200/JCO.2011.41.3807 22869886 PMC3434986

[78] Venstrom JM Zheng J Noor N Danis KE Yeh AW Cheung IY. KIR and HLA genotypes are associated with disease progression and survival following autologous hematopoietic stem cell transplantation for high-risk neuroblastoma. Clin Cancer Res. (2009) 15:7330–4. 10.1158/1078-0432.CCR-09-1720 19934297 PMC2788079

[79] Tarek N Le Luduec JB Gallagher MM Zheng J Venstrom JM Chamberlain E. Unlicensed NK cells target neuroblastoma following anti-GD2 antibody treatment. J Clin Invest. (2012) 122:3260–70. 10.1172/JCI62749 22863621 PMC3428088

[80] Balsamo M Scordamaglia F Pietra G Manzini C Cantoni C Boitano M. Melanoma-associated fibroblasts modulate NK cell phenotype and antitumor cytotoxicity. Proc Natl Acad Sci USA. (2009) 106:20847–52. 10.1073/pnas.0906481106 19934056 PMC2791633

[81] Semeraro M Rusakiewicz S Minard-Colin V Delahaye NF Enot D Vely F. Clinical impact of the NKp30/B7-H6 axis in high-risk neuroblastoma patients. Sci Transl Med. (2015) 7:283ra55. 10.1126/scitranslmed.aaa2327 25877893

[82] Bottino C Dondero A Bellora F Moretta L Locatelli F Pistoia V. Natural killer cells and neuroblastoma: tumor recognition, escape mechanisms, and possible novel immunotherapeutic approaches. Front Immunol. (2014) 5:56. 10.3389/fimmu.2014.00056 24575100 PMC3921882

[83] Ames E Canter RJ Grossenbacher SK Mac S Smith RC Monjazeb AM. Enhanced targeting of stem-like solid tumor cells with radiation and natural killer cells. Oncoimmunology. (2015) 4:e1036212. 10.1080/2162402X.2015.1036212 26405602 PMC4570100

[84] Leung W Iyengar R Turner V Lang P Bader P Conn P. Determinants of antileukemia effects of allogeneic NK cells. J Immunol. (2004) 172:644–50. 10.4049/jimmunol.172.1.644 14688377

[85] Perez-Martinez A Fernandez L Valentin J Martinez-Romera I Corral MD Ramirez M. A phase I/II trial of interleukin-15–stimulated natural killer cell infusion after haplo-identical stem cell transplantation for pediatric refractory solid tumors. Cytotherapy. (2015) 17:1594–603. 10.1016/j.jcyt.2015.07.011 26341478

[86] Ruggeri L Capanni M Urbani E Perruccio K Shlomchik WD Tosti A. Effectiveness of donor natural killer cell alloreactivity in mismatched hematopoietic transplants. Science. (2002) 295:2097–100. 10.1126/science.1068440 11896281

[87] Ruggeri L Mancusi A Perruccio K Burchielli E Martelli MF Velardi A. Natural killer cell alloreactivity for leukemia therapy. J Immunother. (2005) 28:175–82. 10.1097/01.cji.0000161395.88959.1f 15838373

[88] Lundqvist A Yokoyama H Smith A Berg M Childs R. Bortezomib treatment and regulatory T-cell depletion enhance the antitumor effects of adoptively infused NK cells. Blood. (2009) 113:6120–7. 10.1182/blood-2008-11-190421 19202127 PMC2699233

[89] Veluchamy JP Kok N van der Vliet HJ Verheul HM de Gruijl TD Spanholtz J. The rise of allogeneic natural killer cells as a platform for cancer immunotherapy: recent innovations and future developments. Front Immunol. (2017) 8:631. 10.3389/fimmu.2017.00631 28620386 PMC5450018

[90] Vitale M Cantoni C Pietra G Mingari MC Moretta L. Effect of tumor cells and tumor microenvironment on NK-cell function. Eur J Immunol. (2014) 44:1582–92. 10.1002/eji.201344272 24777896

[91] Brandetti E Veneziani I Melaiu O Pezzolo A Castellano A Boldrini R. MYCN is an immunosuppressive oncogene dampening the expression of ligands for NK-cell-activating receptors in human high-risk neuroblastoma. Oncoimmunology. (2017) 6:e1316439. 10.1080/2162402X.2017.1316439 28680748 PMC5486189

[92] Castriconi R Dondero A Bellora F Moretta L Castellano A Locatelli F. Neuroblastoma-derived TGF-beta1 modulates the chemokine receptor repertoire of human resting NK cells. J Immunol. (2013) 190:5321–8. 10.4049/jimmunol.1202693 23576682

[93] Hashimoto O Yoshida M Koma Y Yanai T Hasegawa D Kosaka Y. Collaboration of cancer-associated fibroblasts and tumour-associated macrophages for neuroblastoma development. J Pathol. (2016) 240:211–23. 10.1002/path.4769 27425378 PMC5095779

[94] Zeine R Salwen HR Peddinti R Tian Y Guerrero L Yang Q. Presence of cancer-associated fibroblasts inversely correlates with schwannian stroma in neuroblastoma tumors. Mod Pathol. (2009) 22:950–8. 10.1038/modpathol.2009.52 19407854 PMC3347894

[95] Neviani P Wise PM Murtadha M Liu CW Wu CH Jong AY. natural killer-derived exosomal miR-186 inhibits neuroblastoma growth and immune escape mechanisms. Cancer Res. (2019) 79:1151–64. 10.1158/0008-5472.CAN-18-0779 30541743 PMC6428417

[96] Delgado DC Hank JA Kolesar J Lorentzen D Gan J Seo S. Genotypes of NK cell KIR receptors, their ligands, and Fcgamma receptors in the response of neuroblastoma patients to Hu14.18-IL2 immunotherapy. Cancer Res. (2010) 70:9554–61. 10.1158/0008-5472.CAN-10-2211 20935224 PMC2999644

[97] Felices M Lenvik TR Davis ZB Miller JS Vallera DA. Generation of BiKEs and TriKEs to improve NK cell-mediated targeting of tumor cells. Methods Mol Biol. (2016) 1441:333–46. 10.1007/978-1-4939-3684-7_28 27177679 PMC5823010

[98] Ni LH Tang RN Yuan C Song KY Wang LT Wang XC. FK506 prevented bone loss in streptozotocin-induced diabetic rats via enhancing osteogenesis and inhibiting adipogenesis. Ann Transl Med. (2019) 7:265. 10.21037/atm.2019.05.44 31355232 PMC6614318

[99] Tay SS Carol H Biro M. TriKEs and BiKEs join CARs on the cancer immunotherapy highway. Hum Vaccin Immunother. (2016) 12:2790–6. 10.1080/21645515.2016.1198455 27322989 PMC5137511

[100] Bruenke J Barbin K Kunert S Lang P Pfeiffer M Stieglmaier K. Effective lysis of lymphoma cells with a stabilised bispecific single-chain Fv antibody against CD19 and FcgammaRIII (CD16). Br J Haematol. (2005) 130:218–28. 10.1111/j.1365-2141.2005.05414.x 16029450

[101] Gleason MK Verneris MR Todhunter DA Zhang B McCullar V Zhou SX. Bispecific and trispecific killer cell engagers directly activate human NK cells through CD16 signaling and induce cytotoxicity and cytokine production. Mol Cancer Ther. (2012) 11:2674–84. 10.1158/1535-7163.MCT-12-0692 23075808 PMC3519950

[102] Hu W Wang G Huang D Sui M Xu Y. Cancer immunotherapy based on natural killer cells: current progress and new opportunities. Front Immunol. (2019) 10:1205. 10.3389/fimmu.2019.01205 31214177 PMC6554437

[103] Gauthier L Morel A Anceriz N Rossi B Blanchard-Alvarez A Grondin G. Multifunctional natural killer cell engagers targeting nkp46 trigger protective tumor immunity. Cell. (2019) 177:1701–13 e16. 10.1016/j.cell.2019.04.041 31155232

[104] Rosenberg SA. Immunotherapy of cancer by systemic administration of lymphoid cells plus interleukin-2. J Biol Response Mod. (1984) 3:501–11. 6389777

[105] Sakamoto N Ishikawa T Kokura S Okayama T Oka K Ideno M. Phase I clinical trial of autologous NK cell therapy using novel expansion method in patients with advanced digestive cancer. J Transl Med. (2015) 13:277. 10.1186/s12967-015-0632-8 26303618 PMC4548900

[106] Lupo KB Matosevic S. natural killer cells as allogeneic effectors in adoptive cancer immunotherapy. Cancers. (2015) 11:2019. 10.3390/cancers11060769 31163679 PMC6628161

[107] Rubnitz JE Inaba H Ribeiro RC Pounds S Rooney B Bell T. NKAML: a pilot study to determine the safety and feasibility of haploidentical natural killer cell transplantation in childhood acute myeloid leukemia. J Clin Oncol. (2010) 28:955–9. 10.1200/JCO.2009.24.4590 20085940 PMC2834435

[108] Barry WE Jackson JR Asuelime GE Wu HW Sun J Wan Z. Activated natural killer cells in combination with anti-GD2 antibody dinutuximab improve survival of mice after surgical resection of primary neuroblastoma. Clin Cancer Res. (2019) 25:325–33. 10.1158/1078-0432.CCR-18-1317 30232225 PMC6320320

[109] Tran HC Wan Z Sheard MA Sun J Jackson JR Malvar J. TGFbetaR1 Blockade with galunisertib (LY2157299) enhances anti-neuroblastoma activity of the anti-GD2 antibody dinutuximab (ch14.18) with natural killer cells. Clin Cancer Res. (2017) 23:804–13. 10.1158/1078-0432.CCR-16-1743 27756784 PMC5361893

[110] Modak S Le Luduec JB Cheung IY Goldman DA Ostrovnaya I Doubrovina E. Adoptive immunotherapy with haploidentical natural killer cells and Anti-GD2 monoclonal antibody m3F8 for resistant neuroblastoma: results of a phase I study. Oncoimmunology. (2018) 7:e1461305. 10.1080/2162402X.2018.1461305 30221057 PMC6136849

[111] Federico SM McCarville MB Shulkin BL Sondel PM Hank JA Hutson P. A pilot trial of humanized anti-GD2 monoclonal antibody (hu14.18K322A) with chemotherapy and natural killer cells in children with recurrent/refractory neuroblastoma. Clin Cancer Res. (2017) 23:6441–9. 10.1158/1078-0432.CCR-17-0379 28939747 PMC8725652

[112] Glienke W Esser R Priesner C Suerth JD Schambach A Wels WS. Advantages and applications of CAR-expressing natural killer cells. Front Pharmacol. (2015) 6:21. 10.3389/fphar.2015.00021 25729364 PMC4325659

[113] Hu Y Tian ZG Zhang C. Chimeric antigen receptor (CAR)-transduced natural killer cells in tumor immunotherapy. Acta Pharmacol Sin. (2018) 39:167–76. 10.1038/aps.2017.125 28880014 PMC5800464

[114] Kloess S Kretschmer A Stahl L Fricke S Koehl U. CAR expressing natural killer cells for cancer retargeting. Transfus Med Hemother. (2019) 46:4–13. 10.1159/000495771 31244577 PMC6558329

[115] Tran AC Zhang D Byrn R Roberts MR. Chimeric zeta-receptors direct human natural killer (NK) effector function to permit killing of NK-resistant tumor cells and HIV-infected T lymphocytes. J Immunol. (1995) 155:1000–9. 7608531

[116] Liu E Marin D Banerjee P Macapinlac HA Thompson P Basar R. Use of CAR-transduced natural killer cells in CD19-positive lymphoid tumors. N Engl J Med. (2020) 382:545–53. 10.1056/NEJMoa1910607 32023374 PMC7101242

[117] Li Y Hermanson DL Moriarity BS Kaufman DS. Human iPSC-derived natural killer cells engineered with chimeric antigen receptors enhance anti-tumor activity. Cell Stem Cell. (2018) 23:181–92 e5. 10.1016/j.stem.2018.06.002 30082067 PMC6084450

[118] Esser R Muller T Stefes D Kloess S Seidel D Gillies SD. NK cells engineered to express a GD2 -specific antigen receptor display built-in ADCC-like activity against tumour cells of neuroectodermal origin. J Cell Mol Med. (2012) 16:569–81. 10.1111/j.1582-4934.2011.01343.x 21595822 PMC3822932

[119] Chang YH Connolly J Shimasaki N Mimura K Kono K Campana D. A chimeric receptor with NKG2D specificity enhances natural killer cell activation and killing of tumor cells. Cancer Res. (2013) 73:1777–86. 10.1158/0008-5472.CAN-12-3558 23302231

[120] Parihar R Rivas C Huynh M Omer B Lapteva N Metelitsa LS. NK cells expressing a chimeric activating receptor eliminate MDSCs and rescue impaired CAR-T cell activity against solid tumors. Cancer Immunol Res. (2019) 7:363–75. 10.1158/2326-6066.CIR-18-0572 30651290 PMC7906796

[121] Li Y Liu B Ding S Liu C Chen T Li L. Availability of NK cell expansion agent combined with recombinant IL2 and IL15 stimulation on the expansion and highpurity of NK cells in patients with immunerelated pancytopenia *in vitro*. Mol Med Rep. (2019) 20:4358–66. 10.3892/mmr.2019.10654 31545423

[122] Pillet AH Theze J Rose T. Interleukin (IL)-2 and IL-15 have different effects on human natural killer lymphocytes. Hum Immunol. (2011) 72:1013–7. 10.1016/j.humimm.2011.07.311 21925225

[123] Bi J Tian Z. NK Cell Exhaustion. Front Immunol. (2017) 8:760. 10.3389/fimmu.2017.00760 28702032 PMC5487399

[124] Krneta T Gillgrass A Chew M Ashkar AA. The breast tumor microenvironment alters the phenotype and function of natural killer cells. Cell Mol Immunol. (2016) 13:628–39. 10.1038/cmi.2015.42 26277898 PMC5037278

[125] Liu Y Cheng Y Xu Y Wang Z Du X Li C. Increased expression of programmed cell death protein 1 on NK cells inhibits NK-cell-mediated anti-tumor function and indicates poor prognosis in digestive cancers. Oncogene. (2017) 36:6143–53. 10.1038/onc.2017.209 28692048 PMC5671935

